# Notes on Medical Matters and Medical Men in London

**Published:** 1846-09

**Authors:** David W. Yandell

**Affiliations:** Louisville, Ky.; London


Art. IV.—*Notes on Medical Matters and Medical Men in London.* By David W. Yandell, M.D., of Louisville, Ky.

Mr. Walsh, author of a recent and successful work on *Cancer*, who is now lecturing on *Pathology* at the University College, is one of the best lecturers to whom I have yet listened in London. He is somewhat too rapid, and not sufficiently distinct, but he has life and animation which make up for other defects, keeping one awake while he is lecturing. Dr. Quain, who has distinguished himself by his anatomical works, as a lecturer is above the common run of professors, but neither he, Liston, Murphy, Thompson, nor Williams, of the University, nor Stanley, of St. Bartholomew's would be fancied by the majority of American medical students. Liston is a poor speaker; Cooper is but little better; Graham is considered excessively dull, and Thompson, now delivering a course of lectures on medical jurisprudence, certainly is so.

From all that I have heard, I am satisfied that, in manner, the medical teachers in America are superior to the English.

I saw, some days since, the great "*grinder*," Powers. He is a little round-faced, spectacled man, with an amiable, though not intellectual expression of countence, who, with a strange certainty, prepares candidates for a successful examination before the Apothecaries' Hall. He has now a class of one hundred and twenty, some of whom are taking first lessons; others are more advanced, while a few are being "rubbed up" for the final ordeal to which they expect soon to submit. I was told by a gentleman who passed the Hall not many months ago, that Powers asked him in the morning questions which were propounded to him by the board of examiners in the evening. Whether this was accidental or not he did not express an opinion; I have no doubt it was. The usual fee of the "grinder" is twenty-five dollars, and I believe is never less than this. Some years ago there was another Powers, also a popular "grinder," whose annual income is said to have been not less than £2,000, upon which he amassed such a fortune that he has been able to retire from business.

On the 9th of June I saw Mr. Liston perform several operations, one of which was tying the brachial artery for *false aneurism* of the hand. This is the case of which I made mention in my last letter, as being under treatment, by pressure, in the University College hospital. A young man, while shooting on "good Friday," had the misfortune to burst his gun, which tore off three of the fingers of his left hand, and inflicted an extensive and deep wound on the hand. While the wound was under treatment pulsation was observed in a small, filbert-like tumor situated near the centre of the palm. Mr. Liston advised pressure to the brachial artery, which was applied with very evident benefit, for a season, but after a time the pulsation in the tumor returned, and the patient becoming dissatisfied with the tardy process of cure insisted upon an operation, which Mr. Liston executed with his characteristic neatness and success.

Mr. Murphy I have mentioned as professor of midwifery in the London University, whose writings and practice have placed him in the first rank of English obstetricians. His lectures are full of instruction. I was much interested in one which he delivered a few days since on the causes of *tedious and difficult labor.* He does not regard *hydrocephalus* as so serious an obstacle to labor as it is esteemed to be by many accoucheurs. In the first place it does not follow, he maintains, that the cranium of the child with hydrocephalus is so distended with fluid that compression of the bones cannot be effected; for in many cases where the head was greatly increased in size he has found the bones movable and easily compressed. He cited in proof of his position the six cases of hydrocephalic children reported by Collins, of which only a single one caused a difficult labor. Dr. Robert Lee, however, attended five cases of a similar description in which all gave rise to tedious and difficult labors. Professor Murphy clears up this contrariety in men equally entitled to belief, by saying that Collins saw his cases in a hospital, where, having the management of them from the earliest stage of labor, he was prepared to meet and ward off any untoward symptoms that arose, while Dr. Lee saw his patients after they had been in the hands of midwives who probably did more harm than good, and only called him in to relieve them of their difficulties. If trouble does arise from this cause, it is when the head is at the brim of the pelvis, where various accidents may detain it; but when the pelvic cavity is once fairly attained delivery will take place. Should the head not clear the brim it is your duty to perforate it; the extraordinary size of the fontanels and the peculiar feel of the head rendering the diagnosis easy.

Prof. Murphy judges of the health of a child by the way in which ossification goes on, the age at which dentition begins, and the firmness of the osseous system. He has observed, that one of the obstacles to labor---increased ossification of the bones of the pelvis—is not met with in the upper classes of society, nor among the lower, in cities, but is found in a rustic population, the females of which possess a masculine vigor of constitution, and in whom there is an abundant deposit of bony matter. He has remarked that the opposite cause to this—deficiency of ossific development—is met with in the poor of cities, among females of delicate constitutions, whose systems have never deposited bony materials in a normal quantity.

Prof. M. has been in the habit, for several years, of suggesting in his lectures an operation which has been three times lately performed by Dubois, and is henceforth to rank as one of the legitimate operations in surgery. In cases of contraction of the cervix uteri to an extent rendering labor difficult and dangerous, he proposes that incision into the neck should be practised. For this purpose the knife was used by Dubois, in some of his cases; but in others he deems the scissors preferable. Professor Murphy would be governed in the choice of the instrument by the nature of the case; where the cervix is far back he would use the scissors to relieve the posterior lip, but if it were forward and readily reached, he would resort to the knife. The knife used is the bistoury employed in removing the amygdalæ. The index finger of the left hand is to be introduced into the vagina, placing the palmar surface upon the spot where the incision is to be made; the bistoury is next carried along in contact with the finger until its point, passing within the cervix, comes in contact with the end of the finger. By means of a semicircular motion the cutting edge of the bistoury is then directed perpendicularly towards the free edge of the os tincæ; if this be tense the incision will be most easily effected by a sawing motion of the instrument. Prof. M. advises that the incision be made small, and if one is not found sufficient would make others. If the incision is to be made to the right, the right hand must be used instead of the left. The dilatation is generally sufficient in the course of a quarter of an hour after the operation to use the forceps, if it be desirable to terminate the labor by instruments.

The counter-indications to this operation are, thickening of the cervix, and attachment of the placenta near this part of the uterus. If the cervix has not become thin, the operation by inducing hemorrhage, or causing laceration from the extent of the incision, may terminate fatally instead of proving an advantage.

In his clinical lecture, some days since, Dr. Quain insisted that there is no such thing as purely spasmodic stricture of the urethra. In cases of stricture, after using fomentations to the lower part of the abdomen and perineum, a warm bath, tartar emetic, a saline purgative and opium, he recommends the introduction of a *soft* catheter, instead of the one commonly employed. He is in the habit of using an instrument of this description both in hospitals and his private practice, and he prefers a large catheter, which he has sometimes been successful in introducing after surgeons had failed to pass a small instrument. He can give no directions as to the manner of introducing the catheter, dexterity in this, as in nearly all the operations in surgery, depending upon practice. He advises students to carry a catheter in their pockets, and to take every opportunity to introduce it into the dead subject. Hold the instrument loosely, are his directions, and rather suffer it to pass by its own gravity than use any force to carry it forward. He spoke of two cases of retention of urine, one occurring in a young man in consequence of stricture; the other in the person of a man eighty years of age, produced by enlarged prostate, which, after existing for sometime, brought on paralysis of the bladder. In the young man the bladder could be felt above the pubis, round and well defined; in the man advanced in life this roundness was absent, and the outlines of the distended organ were not well defined. The urine in paralysis of the bladder is muddy, and, under the microscope, purulent. He injects the bladder in such cases with warm water to wash it out, and gradually reduces the temperature of the water until it is cold, which acts favorably in restoring the contractility of that viscus.

Erysipelas is at present prevailing in the University College hospital, where Dr. Quain lectures, but not to a serious extent; it had supervened upon the amputation of a great toe, and had followed a wound of the face. At certain seasons of the year, owing to the constitution of the atmosphere or some other cause, it attains an epidemic prevalence, seizing upon every wounded surface, and following the slightest operations. Dr. Quain has not much confidence in local applications in this affection; it is a constitutional disease and local remedies cannot reach it. When it follows upon wounds, he thinks it well enough to apply fomentations, which answer the good purpose of allaying pain; but as to all the other topical means, he has tried them without much benefit, and believes that *aqua fontana* is about as efficacious as *lotio plumbi*, or indeed anything else. In hospital practice he has abstained from depletory measures, and is in the habit of giving stimulants, the alcoholic drinks to which the patients are accustomed being preferable to wine, which hospital patients seldom or never use.

To-day I saw Mr. Morgan, of Guy's hospital, amputate the fore-arm for diseased carpus. He is a good operator, rather too slow, I should say, and not by any means striking. Guy's hospital was built by Thomas Guy, who left an immense property for its maintenance and support. Being a private institution, that is, not supported by private contributions or by government patronage, it publishes no report of the number of patients annually treated in it, and other statistics of the kind, which you see about the steward's office in the other hospitals. The number of beds averages five hundred, the wards being capable of containing more. Bransby Cooper, Key, and Morgan are the surgeons; Addison, Babington, and Barlow are the physicians, and Oldham and Lever the obstetricians. The school in connection with this hospital I should judge, from the manner in which the students are instructed in going round the wards with the physicians and surgeons, is one of the best in London. Powers, the grinder, I am informed, says "his best men are from Guy's."

The *régime* is different from that of the medical department of the London University; students and teachers are not at the same immeasurable distance from each other; more is said in the wards of the hospitals; more questions are asked by the pupils, and altogether more instruction given and received. There is a good class now in attendance at this school. Students attend four winter courses, and about eighty entries are made, yearly, which would give a class of two hundred and forty or fifty. Some years ago, when only those who had studied in London were eligible to the degree in the College of Physicians and Surgeons, the classes of this and all the other metropolitan schools were larger than at present, students from the provincial schools being now allowed to become candidates.

The museum at Guy's numbers nearly ten thousand specimens, eight thousand of which pertain to physiology and pathology, and the remainder to comparative anatomy.—There are also in the museum three thousand drawings illustrative of the different parts of the human system, besides about seven hundred diagrams. An artist is kept constantly employed in this department, at a salary of $2000 a year, and some of his work is highly creditable. The various forms of cutaneous disease he has represented so faithfully that students are afforded means of recognising and distinguishing between them, such as they will seldom meet with elsewhere. I saw several young men in the museum studying anatomy from the views in wax, which were spread out in glass cases; and a capital mode of study it is too, second only to dissection of the dead body, and, in this hot weather, far preferable to that. You see just in front of you, as you enter this apartment, "Silence is indispensable in a place devoted to study," in large letters. Were I a student at Guy's, I should be tempted to pass much of my time learning anatomy in this quiet, comfortable room.

St. Bartholomew's hospital is one of the largest and wealthiest of all the London charities. In common with all similar institutions here, it has an operating theatre, an anatomical museum, and a library attached to it. The museum contains three thousand specimens; the library has four thousand standard works; the number of its beds is five hundred and thirty, and in 1845 the number of its patients was forty-two thousand, of which five thousand five hundred were "in patients," and the remainder "out patients and casualties." Mr. Lawrence, who is surgeon extraordinary to the Queen is principal surgeon to Bartholomew's. As a writer of great ability Mr. Lawrence is well known in America, and at home he is esteemed one of the best educated and most scientific men in England. He has a fine intellectual face, and his manners are peculiarly bland, cordial and agreeable. Dr. Rigby is also connected with this hospital.

University College hospital is not on so large a scale as either Guy's or St. Bartholomew's. It is one of the hospitals supported by voluntary contributions, and was erected, and is sustained principally for the benefit of the medical college, which is situated immediately opposite to it. Notwithstanding its comparatively small size, it is an institution at which much is to be learned. The number of its beds is about one hundred and fifty, and an addition to it which will soon be completed will make room for fifty more. The number of "in patients" last year was thirteen hundred and seventy-nine; of "out patients" six thousand eight hundred and forty-six; of lying-in women attended at their own residences four hundred and eighteen; of casualties eight thousand three hundred and seventy-nine, making, in all, seventeen thousand and forty. Samuel Cooper, Liston, Quain, Morton, Taylor, Williams, and Murphy are connected with this hospital.

While noticing the public institutions in London connected with our profession I must say something of one of the most magnificent of these, the Hunterian Museum.

John Hunter died on the 16th of October, 1793, aged 65 years. In his will he directed his museum to be offered to the British Government on such terms as appeared reasonable, and in the event of refusal by the government, then to be sold, in one lot, to a foreign State, or such purchaser as might offer. About 1799 Parliament appropriated the sum of fifteen thousand pounds for the purchase of the museum. An offer of the collection was first made by the government to the College of Physicians, but as the College was not wealthy it declined to receive it, and its example was followed by the Royal Society and the British Museum. Finally, it was tendered to and accepted by the College of Surgeons, which has gone on in the great work of which the museum of Hunter was the basis, until it has now a collection unequalled in the world. Mr. Lawrence speaking of the museum says, "so imperfectly was this creation of genius appreciated at that time, that the offer to the government was received very coldly, and six years elapsed before it was finally accepted. When it was mentioned to Mr. Pitt, then Prime Minister, he said, 'What; buy preparations? I have not money to buy gunpowder.'"

The main terms and conditions of the donation were briefly these:—1st. The collection shall be open four hours in the forenoon of two days every week for the inspection and study of the fellows of the College of Physicians, the members of the Company of Surgeons, and persons properly introduced by them; a catalogue of the preparations and an officer qualified to explain it being at such times always in the room. 2d. That one course of lectures, not less than twenty-four in number, on comparative anatomy and other subjects, illustrated by specimens in the museum, shall be given every year by some member of the Company. 3d. That the preparations shall be kept in as good a state of preservation as possible at the expense of the corporation. The museum is open to the fellows and members of the college and such individuals as they may introduce; to members of all the learned and scientific bodies in the United Kingdom; to students of medicine, and to learned and scientific foreigners, members of parliament, peers, &c.

In 1806 and subsequently parliament appropriated £27,-

500, and the College of Surgeons about an equal sum for the erection of an edifice to contain and exhibit the Hunterian collection. This was completed in 1813. The industry of the members of the College was such, that in 1835 the building had become too small to contain the additions which had been made to the collection and the rapidly increasing library, and the College came forward and subscribed £40,000 for the erection of the present immense edifice in which is to be seen the Hunterian and collegiate collections. The College up to this time has expended upon the museum, exclusive of incidental expenses, upwards of £70,000; and the annual charge for its support falls but little short of £3,000.

The Hunterian collection contained, in all, ten thousand five hundred and sixty-three specimens. Of these 963 pertained to osteology, 1345 to natural history, 1215 were fossils, 617 dry preparations, and 3745 preparations in spirits; constituting the *physiological* department. The *pathological* department contained of preparations in spirits 1084 specimens, dry preparations 625, monsters and congenital malformations 218, calculi and concretions 536, microscopic preparations of normal and abnormal structures 217. The members of the College have added 12,347 specimens to the museum; of which, in the physiological department, 2119 are osteological, 240 are dry preparations, 1998 are in spirits, 427 relate to natural history, and 1200 are fossils; and, in the pathological department, the preparations in spirits are 2142, the dry preparations 1365, the monsters and congenital malformations 187, calculi and concretions 884, and the microscopic specimens 1791.

The museum, through the liberality of its proprietors, is open four days in the week, instead of two, and under special circumstances visitors are often admitted on the other two. The library, consisting of works on all branches of medicine as well as the collateral sciences, embracing a great number of most costly books relating to natural history, amounts to 20,000 volumes; it is kept complete by the regular addition of new works, and affords every facility for study, being open from 10 o'clock until 4 daily. The books have cost the College about £10,000, and the annual expense of the library is about £600. The average weekly number of visitors is one hundred and twenty. The Council, some years ago, instituted studentships, three in number, with salaries of £100 per annum, which are held for three years. The appointments are bestowed as the rewards of merit, the test being a strict examination.

The College of Surgeons was incorporated by a charter granted in 1800 by George III. Under her present majesty the charter has been amended, and the institution has taken the name of the Royal College of Surgeons of England. To this corporation the Hunterian museum belongs, and to it is also due the credit of having retained the inestimable "intellectual treasure created by the genius of one of her most gifted sons." The manner in which this trust has been augmented; the liberality with which the doors of the museum and library are thrown open to, I may say, every one; the order and talent displayed in the arrangement of the specimens—where, for example, fossil remains are placed in juxtaposition to the most nearly allied existing species, thus making the vestiges of a former world throw light upon the laws of organization—the excellence of the descriptive catalogues; the memoirs published by different members of the College, all testify in the strongest language to the intelligence, love of science, and indefatigable zeal of the governors of the institution. These are men who, by age and professional eminence, have entitled themselves to the management of its affairs.

It would take up an entire number of your Journal to give even a synopsis of the specimens contained in this mighty receptacle, each one with its label descriptive of what it is. The College is about publishing a catalogue of the pathological cabinet, which will contain, as far as it is known, the history of every patient whose case has furnished specimens to the collection. In this department, among thousands of other interesting things, is the sac of a *popliteal aneurism*, from a man on whom John Hunter operated, at St. George's hospital, in December, 1785. This was the first case in which the operation for popliteal aneurism was performed according to Hunter's new method—tying the vessel on the anterior part of the thigh, at some distance from the diseased point, "thereby to diminish the risk of hemorrhage, and admit of the artery being more readily secured, should any accident happen." This sagacious surgeon was also of the opinion, that "the force of the circulation being thus taken from the aneurismal sac, the progress of the disease would be stopped; and he thought it probable that, if the parts were left to themselves, the sac with its contents might be absorbed, and the whole tumor be removed, which would render an opening in the sac unnecessary." The history of this interesting case is as follows:

The patient was a coachman, aged 45 years; the disease had first been perceived three years previous to his admission into the hospital, and had gradually increased during the whole of that period. He recovered from the operation and returned to his employment, but died from the effects of fever fifteen months afterwards. On examination after death, the cicatrix on the anterior part of the thigh was scarcely perceptible; the ham had no appearance of tumor, and, to the eye, was exactly like that of the other limb; but there was a solid tumor perceptible to the touch filling the hollow between the condyles. The femoral artery was impervious from the point where it gives off the arteria profunda as low down as the portion included in the ligature; and at that part there was an ossification for an inch and a half along the course of the artery, of an oval form, the rim of which was solid, becoming thinner towards the centre, and not bony but ligamentous. Below this part, the femoral artery was pervious down to the aneurismal sac, and contained blood, but did not communicate with the sac itself, having become impervious just at its entrance. What remained of the aneurismal sac was somewhat larger than a hen's egg, but more oblong and somewhat flattened, extending along the artery a little way; it was perfectly circumscribed, not having the smallest remains of the lower orifice of the popliteal artery, and contained a solid coagulum adhering to its internal surface, and which appeared to be composed of concentric laminæ, uniform in color and consistence.

In the case containing urinary calculi, I observed one taken after death from the bladder of Sir Walter Ogilive, which weighed forty-four ounces Troy, and measured sixteen inches around its longer axis, and fourteen around its shorter. Dr. Powell analysed this calculus, and found it to consist of "the triple phosphate of ammonia and magnesia, with phosphate of lime, and a large quantity of animal matter."

I will mention only one other specimen—a tibia and fibula with a large osseous tumor attached. The tumor has grown almost entirely from the anterior and lateral parts of the upper two-thirds of the tibia; it is of an irregularly oval form, and measures ten inches downwards, about fourteen inches from side to side, and exactly one yard in its chief circumference. Its surface is for the most part smooth, and evenly covered by a very thin layer of compact osseous tissue. Its interior is constituted of coarse cancellous tissue, about as heavy as healthy bone.. Its exterior is continuous with the surface of the tibia, the walls of which are expanded and drawn out at its base of attachment. The fibula is pushed outwards, and so compressed by the growth of the tumor, that it is in some parts nearly two inches in width, and hardly two lines in thickness. The limb was amputated at St. Bartholomew's hospital, and weighed, with the foot and its appendages, forty-two pounds. One never grows weary of wandering through this vast repository, where so much is to be seen illustrative of healthy structure, as well as of the ravages of disease; but I fear I have already wearied your readers with these details.

Every scientific traveler in London is attracted to the Royal Institution, not only as the theatre of Sir Humphry Davy's great achievements in chemistry, but as the place where Michael Faraday is now engaged in lecturing. We are familiar with the name of Faraday at home, and I was impatient to hear him in his laboratory. I believe I cannot interest you more than by giving you some account of the institution to which he belongs, and of one of his remarkable lectures.

The Royal Institution of London, a charter for which was obtained in 1800 and confirmed ten years afterwards, possesses a laboratory for the promotion and advancement of chemical knowledge by original investigations and courses of practical lectures, a large library, and a museum, containing a mineralogical collection, composed chiefly of British specimens. The professorships of chemistry are at present occupied Mr. Brande and Dr. Faraday; Mr. John Lindley holds the chair of botany, and Dr. W. B. Carpenter that of physiology. The chair of natural and experimental philosophy is at this time vacant. Everybody, I suppose, has his notions of what constitutes a lecturer; according to my own, Faraday is, beyond all question, the best to whom it has been my fortune ever to listen. If rapidity without haste, fluency without verbiage, succinctness and earnestness, clearness and propriety of enunciation, and ease and grace of manner, are the attributes of an accomplished lecturer, then he is perfect. He is in size rather below the common stature, and, I should say, about fifty years old. The expression of his face is strikingly intelligent. He manipulates with the confidence, dexterity, and precision of a juggler, reminding one forcibly of feats by sleight-of-hand; such is the quickness and perfection of his experiments. I was told by an intelligent gentleman, who sat by my side, a week ago, listening to the last lecture of his present series, that he rarely ever fails in an experiment. The evening on which I heard him was occupied chiefly with illustrations of the *cohesive force of water*. He first spoke of water in its three conditions of solid, liquid, aeriform. Substances which appear perfectly liquid, and which would remain so for an indefinite length of time, if left in a perfectly quiescent state, become solid by the slightest mechanical agitation of their particles. This he illustrated by the well known experiment of a solution of the sulphate of soda, which becomes instantly a crystalline mass on the slightest agitation, or by dropping into it a solid body, or simply pouring it from one vessel into another; but owing to the high temperature of the room this experiment was less successful than I have generally seen it.

Dr. Faraday alluded to the somewhat analogous condition in liquids and vapors, which was discovered by M. Cagnard de la Tour, and is known by his name. It is this:—under a sufficient pressure, and at a certain temperature, some liquids, and water among the rest, may be converted into gases which occupy no more space than the liquids themselves. The slightest attention to the properties of water will convince any one of the error of the common notion, that its particles possess no cohesive force. If you immerse a solid body in water, a portion of the water adheres to it; and this has been called by Dr. Daniell heterogeneous adhesion. But since only particular portions of the water can come in contact with the surface of the solid body, it follows that this adhesion can exist only between it and a mere film of water; and from their ability to resist the power of gravity it is obvious that the accumulated globules must be held together by a strong cohesive attraction between themselves. Numerous experiments may be performed to show this. Thus, by balancing a smooth glass plate, with a known area, at the end of a scale beam, and bringing this into perfect contact with the surface of some water, which is done by covering the glass with a thin pellicle of soap, it will be found that more than an equivalent weight is required to lift the glass, and that a mass of water is actually raised up before the plate is detached. Laplace conjectured, though, as Dr. Faraday holds, erroneously, that the force of cohesion between the water and the glass was indicated by the weight required to raise the plate. As only a thin film of water is in actual contact with the plate, only a small portion of its cohesive force is exerted. The weight required to lift the plate from the water is, in fact, equivalent to the cohesive force existing among the particles of water themselves. Dr. Faraday did not mention the exact force necessary to lift a plate having an area of twenty-five inches, but the number of ounces is surprisingly great.

M. Henry has made the discovery that the boiling point of water may be raised from 212°, its ordinary boiling heat, to 275°, simply by so treating it that all its air shall be expelled. Were it not for the air contained in water, this fluid would not boil at 212°; and when ebullition did take place, the boiling would be like that of concentrated sulphuric acid, turbulent and attended with no little danger. It is therefore the air always present in ordinary water which renders the boiling process tranquil and safe. When the boiling point is raised to 275°, the temperature is equivalent to a pressure of three atmospheres. It is unquestionably the cohesive force of the particles of water among themselves that enables them to resist so high a temperature. This same force we see continually exerted in all those cases in which the liquid is repelled by the surface of solid bodies, where we find that it uniformly assumes the shape of globules. The dew is seen adhering to the grass and leaves in "pearly drops," globular in form.

Dr. Faraday still further illustrated the subject of cohesion by pouring water into a small cylindrical vessel composed of copper wire-gauze. The water remained in the basket as though it were not perforated by countless holes until, by shaking, the force of cohesion was overcome by the power of gravitation. A cup of wire-gauze, with very fine meshes, was next heated to a red heat, and some water poured into it; so long as the heat was preserved the water rolled over the surface, but the moment the temperature was reduced, the water began to escape through the meshes, forming, at the same time, a large quantity of steam. The lecturer alluded to the fact long known, that water may be kept upon a very hot surface without boiling, or generating steam in any appreciable quantity. He poured some water on a plate raised to a white heat, and it rolled about as a globule of quicksilver would do, producing, so far as the eye could discover, no steam. This water was prevented, by a stratum of dry steam, from coming in contact with the heated surface, and had a temperature really below that of boiling and only 105° Fahrenheit. By cooling the surface to a certain point, contact between it and the water is induced, and then the latter passes off in volumes of vapor. Dr. F. substituted sulphuric ether for water, and the same result was obtained, except that the effect was more brilliant, from the ignition of the ethereal vapor, in the midst of the flame of which a globule of the liquid was seen dancing over the heated surface, but not actually touching it. When the vessel was somewhat cooled, the ether coming in immediate contact with it, a much larger quantity of vapor was formed, and an increase in the size of the flame took place, showing even more clearly than the former experiment, that at a very high temperature the fluid is repelled from the surface of the heated plate. This is termed the *spheroidal state* of liquids, and it is on this principle that Boutigny *froze* water in a *red-hot* crucible.

Dr. Faraday repeated this experiment in the following manner:—into a red-hot crucible he poured a quantity of anhydrous sulphurous acid, to which he added subsequently an equal quantity of water. According to the law to which I have just alluded, no contact could exist between the liquids and the heated surface, and the sulphurous acid, boiling very rapidly at a temperature of 14° Fahrenheit, produced so much cold as to freeze the water. The water was hardly added before a lump of ice was turned out of the crucible, the water being frozen by the boiling of the acid. Anhydrous sulphurous acid becoming spheroidal at so low a temperature, it is not necessary to heat the crucible very hot, since the temperature of all liquids in the spheroidal state is a few degrees below their boiling point.

The last, and by far the most brilliant experiment of the evening, was one which the learned professer performed for the first time before the public on that occasion, and consisted in freezing *mercury* in a red-hot crucible. This he effected by the following simple arrangement:—having heated a platina crucible to the requisite temperature he dropped into it a few lumps of carbonic acid, and added a quantity of sulphuric ether; thus he obtained a freezing mixture in a spheroidal state, equal in the air to a cold of 160° below the zero of Fahrenheit. The ethereal vapor was not inflamed, although the crucible was red-hot. A ladle, containing a globule of mercury, was now immersed in this mixture, and in a short time taken out with the quicksilver as solid as a leaden bullet. A more beautiful experiment one could hardly hope or desire to see. Very many of Dr. Faraday's intelligent looking audience were ladies, who during the evening manifested the liveliest interest both in the remarks and the experiments of the professor. I felt myself surrounded by the pure, the refined, and the great of London, and shall long remember the evening as one of the most delightful of my life. I saw Mr. Brande, a fine looking gentleman, and many other distinguished *savans* in the auditory.

I saw, yesterday, an examination of the body of a man whose case I had watched in the hospital with unusual interest. The patient, 42 years old, had suffered for nine months with difficulty in voiding his urine; his general health was gone; his physicians had diagnosed a lumbar abscess of very large size pointing towards Pourpart's ligament.

I was present when Dr. C, J. B. Williams, after a careful and acute examination, pronounced his disease an abscess of the kidneys. In a week or two afterwards he commenced expectorating pus, and died in a few days. The *sectio cadaveris* revealed an abscess of the left kidney, by which the whole of that organ, except a small part of its cortical substance, had been destroyed, and a lumbar abscess which involved more than one-third of the great psoas muscle. The matter from the kidney had made its way along the vertebral column, through the diaphragm, and into the lungs, from which it was freely expectorated.

Mr. Ferguson, of King's College, is the most formidable rival Mr. Liston has in London. Like Liston he is by birth a Scotchman, and like him of a fine personal appearance, with an intelligent face, and great suavity of manners. He is about 38 years of age, and is in a fair way to attain greater eminence as a surgeon than he has already won. But it is as an operating and not as a lecturing surgeon that he is acquiring distinction; his powers as a lecturer would never raise him above mediocrity.

The London Lancet is out upon Dr. Elliotson with almost savage severity. As it will be sometime before you receive your copy, in order that you may see how doctors on this side of the great waters write concerning one another, I will give you an extract from an article which I read in a late number of that Journal.

"After a disappearance from University College and a long submergence in the depths of quackery, the mesmerist (Dr. Elliotson) made his appearance on the professional stage on Saturday last. He rises again to the surface not like the diver with pearls in his hands, but with the mud and weeds of the lowest depths about his brow—a by-word, a hissing and a reproach to his brethren. Does he himself treat the harlotry which he dares to call science with respect? Let the profession consider his allies and assistants, taken from the pert folly of the nobility,—the weakest among the literary people, high and low ladies, quack clergymen, itinerant lecturers and exhibiting buffoons. Look at that more than all-damning fact in the cases of this man, his violation of the sacredness of death itself, when he took the O'Keys to the beds of the dying in the University College hospital, and tortured them at the awful moment of dissolution by the half frenzied half hysterical ravings of these tricksters. And this is the man who dares institute comparisons between his own nothings and the great deeds of Harvey!"

Within a few days I have seen the thermometer in the shade as high as 93°. This would be accounted hot weather anywhere.

London, June 25th, 1846.

